# Combining Desirable Traits for a Good Biocontrol Strategy against *Sclerotinia sclerotiorum*

**DOI:** 10.3390/microorganisms10061189

**Published:** 2022-06-09

**Authors:** Daphné Albert, Tim Dumonceaux, Odile Carisse, Carole Beaulieu, Martin Filion

**Affiliations:** 1 Saint-Jean-sur-Richelieu Research and Development Centre, Agriculture and Agri-Food Canada, Saint-Jean-sur-Richelieu, QC J3B 7B5, Canada; daphne.albert@agr.gc.ca (D.A.); odile.carisse@agr.gc.ca (O.C.); 2 Saskatoon Research and Development Centre, Agriculture and Agri-Food Canada, Saskatoon, SK S7N 0X2, Canada; tim.dumonceaux@agr.gc.ca; 3 Département de Biologie, Université de Sherbrooke, Sherbrooke, QC J1K 2R1, Canada; carole.beaulieu@usherbrooke.ca

**Keywords:** *Sclerotinia sclerotiorum*, biocontrol, antibiosis, induced systemic resistance, mycoparasitism, hypovirulence

## Abstract

The fungal pathogen *Sclerotinia sclerotiorum* (Helotiales: Sclerotiniaceae) causes white mold, a disease that leads to substantial losses on a wide variety of hosts throughout the world. This economically important fungus affects yield and seed quality, and its control mostly relies on the use of environmentally damaging fungicides. This review aimed to present the latest discoveries on microorganisms and the biocontrol mechanisms used against white mold. A special focus is put on the identification of biocontrol desirable traits required for efficient disease control. A better understanding of the mechanisms involved and the conditions required for their action is also essential to ensure a successful implementation of biocontrol under commercial field conditions. In this review, a brief introduction on the pathogen, its disease cycle, and its main pathogenicity factors is presented, followed by a thorough description of the microorganisms that have so far demonstrated biocontrol potential against white mold and the mechanisms they use to achieve control. Antibiosis, induced systemic resistance, mycoparasitism, and hypovirulence are discussed. Finally, based on our actual knowledge, the best control strategies against *S. sclerotiorum* that are likely to succeed commercially are discussed, including combining biocontrol desirable traits of particular interest.

## 1. The White Mold Disease

The fungal pathogen *Sclerotinia sclerotiorum* (*Sclerotinia sclerotiorum* (Lib.) de Bary: kingdom Fungi, phylum Ascomycota, class Discomycetes, order Helotiales, family Sclerotiniaceae, genus *Sclerotinia*.) causes white mold, a disease that can develop during the growing season, as well as in the post-harvest period [1]. Host plants for *S. sclerotiorum* include at least 64 families, 225 genera, and 361 species [2]. White mold has been associated with substantial losses in Australia, Europe, Africa, India, and North America in a wide variety of hosts, mostly Dicotyledonae plants in the Solanaceae, Cruciferae, Umbelliferae, Asteraceae, Chenopodiaceae, and Leguminosae families and a few Monocotyledonae in the Amaryllidaceae and Liliaceae families [1,3,4,5,6,7]. The most important commercial crops affected are canola, soybeans, green beans, lettuces, and carrots [5,8]. The wide host range limits control methods since the number of non-host crops available for crop rotation is limited. Therefore, *S. sclerotiorum* causes significant economic losses of up to several millions of dollars worldwide each year by attacking different plant parts, including stems, leaves, flowers, and fruits, leading to reduced plant yield and quality [3,9,10]. More than $200 million in annual losses associated with white mold has been reported in the United States since 2000 [11,12]. Other phytopathogenic members of the genus *Sclerotinia* can also cause rots, such as *Sclerotinia minor* and *Sclerotinia trifolium*, but these are not as important as *S. sclerotiorum* [13].

The symptoms caused by *S. sclerotiorum* vary to some degree with the host, infection pathways and with the environmental conditions. The fungus can infect the aerial parts of crops and causes flower blights, stem rots, fruit rots, and head blight. Aerial infections are important, considering that the fungus can release millions of spores that are wind-dispersed. On the other hand, *S. sclerotiorum* can also infect plant roots and/or crowns. The fungus penetrates the host cuticle by mechanical pressure [2]. An enzymatic process affects the lamella between cells, which disorganizes the tissues rapidly following penetration [2]. Thereafter, symptoms may differ among host crops, but there are a number of similarities. The most shared symptoms are light-brown or greyish-white, water-soaked spots that develop on leaves and stems, and the formation of a white, cotton-like mycelium on leaves, stems, and petioles [3,6]. Secondary symptoms that appear as the fungal activity progresses are water-soaked lesions, wilting, as well as the bleaching and shredding of plants parts [3,6].

In this review, *S. sclerotiorum*’ disease cycle and its main pathogenicity factors is presented followed by a thorough description of the different microorganisms and biocontrol mechanisms that have so far been used to control white mold and *S. sclerotiorum* under different conditions, ranging from in vitro to the field. The focus is put on the identification of desirable biocontrol traits, the pathogen’s life cycle stages that are targeted, and the conditions required for efficacy. Novel research avenues are also discussed to promote the control of white mold in the field.

## 2. *S. sclerotiorum* Life Cycle

**Figure 1:**
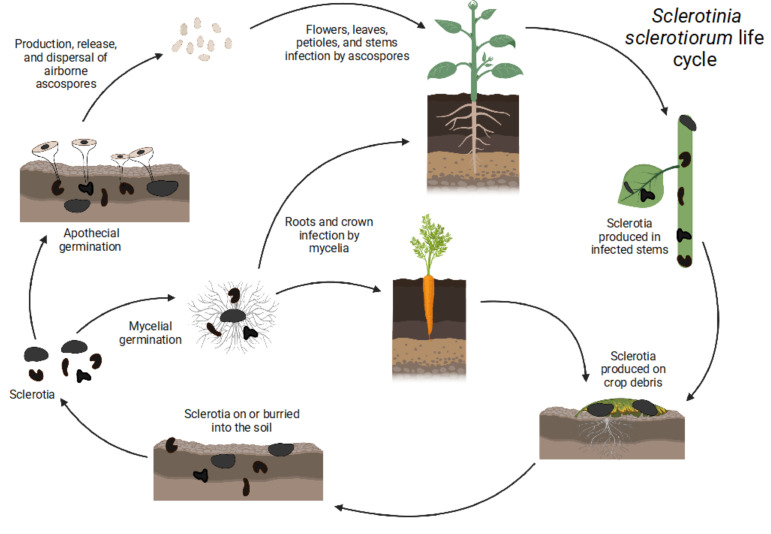
*Sclerotinia sclerotiorum* life cycle: Carpogenic and myceliogenic germination of sclerotia.

The life cycle of *S. sclerotiorum*, shown in Figure 1, is monocyclic, as there is only one cycle of inoculum produced (ascospores). No secondary inoculum or asexual spores (conidia) are produced by *Sclerotinia* species. *S. sclerotiorum* has developed several physiological and developmental strategies for its dispersal, propagation, and survival [14]. The fungus can act as an aerial and subterranean pathogen using sclerotia, which are generally resistant to physical, chemical, and biological degradation [6]. Sclerotia are overwintering structures that can be found inside or outside of the affected plant, mainly on plant debris [3].

The sclerotia consist of three distinct layers, a thick-walled pigmented rind, a thin-walled cortex, and a white medulla [15]. There are three types of sclerotia formed by *S. slecrotiorum*: normal black sclerotia, abnormal black sclerotia, and brownish-tan sclerotia. Tan sclerotia do not produce as much melanin as the two other types, resulting in a lighter color [8]. Studies have shown that sclerotia with a melanized cell wall and an intact rind increase the survival of *S. sclerotiorum* under unfavorable environmental conditions [8]. Abnormal sclerotia are more prone to microbial degradation due to the fragmentation of their rind, which allows nutrients to escape [8]. *S. sclerotiorum* mycelium found in infected stubbles can also overwinter in some areas, but rapidly loses viability in the spring and early summer [8]. Therefore, they are not considered an important source of inoculum for plant infection [8]. With sclerotia, both sexual (carpogenic germination) and asexual (myceliogenic germination) reproduction can occur [6].

## 3. Carpogenic Germination

Aerial infection, also called carpogenic germination, occurs when apothecia, fungal structures containing the ascospores used for dispersal and infection, are produced by the sclerotia [3]. *S. sclerotiorum*’s carpogenic germination depends on three main factors: (1) the geographic origin of the isolates, (2) the temperature at which sclerotia were formed, (3) and the temperature at which the parent inoculum, mycelia, or sclerotia was produced [8]. To overcome dormancy and to germinate carpogenically, sclerotia should be conditioned at low temperatures [6]. The most favorable temperature condition range is from 10 to 20 °C [6]. It has been shown that at temperatures higher than 26 °C, there is no production of apothecia [16], while 21 °C is the ideal temperature for their production [15]. Another important factor of carpogenic germination is moisture [6,15]. The soil water potential should be higher than 100 kPa [15]. Sclerotia that are present in an dry environment are unable to germinate carpogenically [17].

## 4. Myceliogenic Germination

Unlike carpogenic germination, underground infection, also known as myceliogenic sclerotia germination, results in the germination of the mycelium directly from sclerotia, which may subsequently attack plant tissues using enzymes or mechanical force if penetration is not achieved through natural openings [1,3]. The degree of deposition of melanin black pigments both outside and inside the bark cell walls is associated with myceliogenic germination [8]. Myceliogenic germination of black sclerotia occurs in the presence of exogenous nutrients [8]. However, in the absence of exogenous nutrients, myceliogenic germination only occurs when the sclerotia are devoid of black pigments, as in the case of immature sclerotia [8]. Myceliogenic germination is also triggered when normal black sclerotia with crusts are damaged by mechanical means, desiccant treatments, or freezing [8]

## 5. Pathogenicity Factors

The main pathogenicity determinants of *S. sclerotiorum* are hydrolytic enzymes and the production of some metabolites that act as toxins, mainly oxalic acid [1]. The degradation of the plant cell wall, its components, and tissue maceration is achieved through the action of several extracellular lytic enzymes [9]. The production of cell-wall-degrading enzymes (CWDEs) facilitates plant colonization. CWDEs include pectinases, β-1,3-glucanases, glycosidases, cellulases, xylanases, cutinases, and redox enzymes that are involved in lignin modification [9,18]. The flexibility of the pathogen to colonize its host is facilitated by its broad array of CWDEs, which is reflected in a variety of isoelectric points and molecular weights, along with differential transcriptional regulation [9].

### 5.1. Cell-Wall-Degrading Enzymes (CWDEs)

The expression of most CWDE-encoding genes is regulated at the transcriptional level by the availability of carbon and/or nitrogen [1]. The fungus can adapt its metabolism according to the presence of glucose and/or other available carbon sources that can be metabolized due to the repression of the carbon catabolism [1], in a manner similar to white-rot Basidiomycetes, which are primarily saprophytic [19]. Ambient pH can also regulate CWDEs’ production at the transcriptional level [1]. The penetration of the fungus inside the plant and the maceration of the tissues is facilitated by pectinases. Pectin is a major component of plant cell walls [6]. Penetration and colonization of the host is facilitated by the hydrolysis of pectin, by weakening the cell wall structure [1]. This process provides a carbon source for the growth of the fungus [1]. Several forms of pectinolytic enzymes capable of killing plant cells are produced by *S. sclerotiorum* and the expression of their encoding genes is pH dependent [1,6]. A study has shown that a pH of 4–5 is ideal for pectinolytic enzyme production [6].

### 5.2. Oxalic Acid

Oxalic acid has different functions in fungi. It plays a role in pathogenesis, controls nutrient availability, regulates soil chemistry such as Ca^2+^ levels, detoxifies copper compounds, and degrades plant lignocellulose [20]. It is well known that oxalic acid contributes to the pathogenicity of many necrotrophic fungi. This compound may play different roles during the infection process [21]. As mentioned earlier, monocot plants are less infected by *S. sclerotiorum*. Most monocots produce oxalate oxidases (members of the oxidoreductase family) that catalyze the conversion of oxalic acid into H_2_O_2_ and CO_2_ [7]. A study has shown that fungal mutants not producing oxalic acid had lost their pathogenicity, leading to the conclusion that oxalic acid is a required factor for pathogenicity [21]. Oxalic acid affects the accumulation of potassium and the hydrolysis of starch in guard cells (two conditions necessary for the opening of the stomata) as well as disrupts the abscisic acid (ABA)-dependent process that leads to the closure of stomata [21]. Oxalic acid forms reactive oxygen species (ROS), which suppress the oxidative breakdown that serves as a defense mechanism of the front line of host defenses [21]. Similarly, some lignin-degrading white-rot Basidiomycetes produce oxalate as a means of generating ROS through the activity of extracellular enzymes such as manganese peroxidase and lignin peroxidase, which degrade oxalate to produce formate anion radicals that are quickly oxidized to produce superoxide and ultimately H_2_O_2_ [22,23,24,25]. These ROS support not only lignin oxidation, but additionally, through the activity of enzymes such as cellobiose dehydrogenase, they initiate the degradation of cellulose by a combination of oxidative and hydrolytic mechanisms [19,26,27]. The recovery of oxalic acid from infected tissues, the correlation between the quantity produced and the severity of the disease, and the development of symptoms (formation of lesions and water-soaked tissues) after the direct plant application of oxalic acid suggest that the production of oxalic acid is a key element of Sclerotinia’s pathogenicity [1]. Xu et al. reviewed the mechanisms/molecules involved in *S. sclerotiorum* virulence and concluded that oxalic acid is an essential contributor. However, they showed that disease development was mostly correlated with acidic pH and not with oxalic acid production per se. Mutants that lost their ability to produce oxalic acid but that accumulated fumaric acid were still able to cause disease development on different plants. Additional analyses also suggested the existence of an unrecognized acid-responsive regulator [28]. A new research avenue investigating pH sensing/regulation in *S. sclerotiorum* to identify this unknown pH regulator could provide new targets to control this pathogen.

## 6. Plant Disease Resistance against White Mold

It is difficult to control *S. sclerotiorum* regardless of which plant it infects because of the long-term persistence of sclerotia in soil, its ability to produce ascospores that can be wind-dispersed, and its ability to adapt to control measures [6,8,29]. When the climate and management practices favor a high yield potential, such as through a dense canopy and/or irrigation, white mold can significantly affect plants [30]. The level of natural plant resistance against *S. sclerotiorum* is low, making the disease caused by this pathogen very difficult to control [31]. The different aspects of plant resistance have been recently reviewed by O’Sullivan et al. and Wang et al. Numerous efforts have been and are still being made to develop sclerotinia-resistant crops. Conventional and molecular breeding relying on natural sources of resistance has so far been challenging because of the multiple minor genes that contribute resistance against *S. sclerotiorum* [13]. Recent progress made by Wang et al. has broadened our understanding of the genetic architecture underlying the quantitative resistance to *S. sclerotiorum,* by describing candidate resistance genes and classifying these potential gene targets with regards to their implications in different stages of the defense process. However, although plant genetic control against *S. sclerotiorum* has been under development for many years now, breeding programs have not yet provided significant resistance to *S. sclerotiorum*. Therefore, the most popular methods to control white mold remain chemical and cultural control approaches [8].

## 7. Disease Control Using Fungicides

To this day, the use of fungicides is the most effective means of controlling *S. sclerotiorum* [3]. While foliar-applied fungicides can be effective against the fungus, none provide complete control under all conditions [3]. Control is often inconsistent, mainly due to difficulties in obtaining good fungicide distribution, coverage and application timing in relation to the release of ascospores, as well as the increased disease pressure, the emergence of resistance, and problems associated with the rapid microbial degradation of fungicides present in the soil [32,33]. The main active fungicidal ingredients against *S. sclerotiorum* are: boscalid, fluazinam, fluxapyroxad, pyraclostrobin, penthiopyrad, picoxystrobin, prothioconazole, trifloxystrobin, tetraconazole, and thiophanate methyl [6]. When applying fungicides to control *S. sclerotiorum*, key elements must be considered to provide significant results, including the level of moisture observed over the past few weeks prior to application for apothecia development and survival, the canopy thickness and the yield potential, the weather forecasts for the week to come, and the number of pathogens present [6].

The most commonly used fungicides to eradicate white mold are benzimidazoles and dicarboximides [34]. However, *S. sclerotiorum* populations have become resistant to most fungicides due to their widespread use. Several countries now report *S. sclerotiorum* strains that are resistant to benzimidazoles and dicarboximide [34]. New fungicides with novel modes of action based on thiazolidine compounds containing nitrogen and sulfur are being investigated to minimize the losses caused by white mold [34]. As most synthetic pesticides can be harmful to humans and the environment, more environmentally friendly alternative methods are being sought to reduce their use [6].

## 8. Physical and Cultural Control Methods

Considering that sclerotia are produced on crop debris, they should be tilled into deep layers of the soil to prevent sclerotia from germinating. However, this method will only be effective in the short term (one season) since next-season cultivation will bring new sclerotia to the soil surface that will be able to germinate. Flooding soils for 2 to 3 weeks with irrigation significantly reduces the viability of sclerotia, however, this practice may not be applicable to all crops and production areas [35]. The density of plants must be chosen so that it does not create a microclimate (high humidity for prolonged periods) favoring the germination of sclerotia. In some cases, it is possible to choose cultivars with erect foliage to avoid crowding of the foliage, which impairs the good airflow required to reduce high moisture within the canopy. For example, carrot foliage trimming was proven to be very effective at reducing white mold without affecting the marketable yield [36]. Humidity plays an important role in the development of white mold. Prolonged tissues wetness is essential for ascospore infection, while moist soils favor mycelium infection. Irrigation, when needed, should be done early in the morning to allow enough time for the plant tissues to dry during the day. Drip irrigation should be used whenever possible. Long-term crop rotation with non-host crops such as corn or wheat can help reduce the stock of viable sclerotia in the soil.

## 9. Biocontrol

Alternative control methods exist, such as biological control or biocontrol [37]. Biocontrol is a method that controls plant diseases through the use of beneficial microorganisms or microbial metabolites [37] that have harmful activity against pathogens and/or the diseases they cause [37]. There are several organisms that can be used as biocontrol agents, mainly fungi and bacteria, which rely on various direct and indirect mechanisms to protect the plant from the pathogen such as antimicrobial metabolite production (antibiosis), the stimulation of the plant’s disease resistance mechanisms, parasitism, and hypovirulence.

## 10. Biocontrol Mechanisms against *S. sclerotiorum*

### 10.1. Protection through Antibiosis

Some antagonistic bacteria and fungi secrete extracellular antimicrobial metabolites (mostly antibiotics) that are inhibitory at low concentrations against plant pathogens [38,39]. The modes of action of the various antibiotics produced by such phytobeneficial microorganisms can be very different, ranging from altering the cell membrane to having inhibitory effects on key cellular constituents such as ribosomes [39]. Antibiotics produced by phytobeneficial microorganisms include compounds such as 2,4 diacetylphloroglucinol, phenazine-1-carboxylic acid, phenazine-1-carboxamide, pyoluteorine, pyrrolnitrine, butyrolactones, kanosamine, zwittermycin-A, rhamnolipids, cepaciamide A, pseudomonic acid, and cepafungins, which display antifungal effects [39]. Several studies have specifically demonstrated, with the use of knock-out mutants or with confrontation tests using pure isolated antibiotics, the action of specific antimicrobial compounds against *S. sclerotiorum*. These results are summarized in Table 1.

Among plant-beneficial bacteria producing antimicrobial compounds of biocontrol interest, *Bacillus* sp. have been widely studied [50]. Many *Bacillus* spp. produce cyclic lipopeptides (LP), including surfactin, iturin, and fengycin, that display various activities [50]. The lipopeptide surfactin has strong surface and biological activity that includes emulsifying and foaming properties [37]. At certain dosages, it anchors in the lipid layer and damages the integrity of biofilms [37]. Among iturins, iturin A and C, bacillomycin D, F, L, LC, and mycosubtiline are the main variants of this chemical family that increase membrane permeability and therefore display strong antifungal activity [37]. This antifungal activity is based on the formation of pores in the fungal cell membranes that cause an osmotic imbalance, membrane disruption, and solubilization [51]. The antimicrobial effect of fengycin is high, especially against filamentous fungi [51], as this molecule interacts with the membrane lipid bilayer. It causes its solubilization, modifies its structure and permeability, and creates ion-conducting channels that pass through it [37,51]. The co-production of surfactin, fengycin, and iturin is responsible for strong antifungal activity [51]. Among *Bacillus* sp., *B. amyloliquefaciens* is an excellent example of a biocontrol agent that displays strong antifungal activity against *S. sclerotiorum* [37]. It produces different LPs, such as surfactins, fengycins, and iturins [37]. Farzand et al. demonstrated that the *B. amyloliquefaciens* strain EZ1509 displays antifungal activity in in vitro confrontational assays against *S. sclerotiorum,* leading to ultrastructural changes in hyphae. The presence of surfactins, iturins, and fengycins was confirmed by MALDI-TOF-MS in the inhibition zone, suggesting their implication in the inhibition [51,52]. Tests on detached tobacco and rapeseed leaves were also performed to demonstrate the biocontrol antagonism effect of this strain [51]. The production of the cyclic LP fengycins, iturins, and surfactins by EZ1509 was again pointed as being responsible for the modified ultrastructure of fungal membranes, leading to the leakage of cellular metabolites and ultimately cell death.

Many *Pseudomonas* sp. have also been studied for their biocontrol activity against *S. sclerotiorum*. The *P. chlororaphis* strain PA23 has demonstrated antifungal activity in vitro and in greenhouse assays on lettuce against *S. sclerotiorum* [42]. PA23 secretes the antibiotics pyrrolnitrin and phenazines in addition to producing hydrogen cyanide (HCN). Selin et al. demonstrated that pyrrolnitrin is the primary antibiotic for the biocontrol activity of PA23 and that phenazines play a role in facilitating biofilm formation. HCN contributes to the overall antifungal activity of PA23 against *S. sclerotiorum* [42]. Experiments were performed in growth chambers, where the contribution of HCN produced by PA23 against *S. sclerotiorum* was tested by monitoring three symptoms that are associated with the fungus: (i) crown rot, (ii) stem rot, and (iii) leaf discoloration [42]. Plants treated with isogenic mutants of PA23 not producing HCN developed crown and stem rot when compared to plants treated with wildtype PA23, where no disease symptoms were observed [42].

Many *Streptomyces* sp. have also been studied for their potential to be used as biocontrol agents [53]. They are known for their ability to produce a variety of bioactive antimicrobial compounds [53,54]. A recent study showed the antifungal activity of the antibiotic wuyiencin produced by *Streptomyces albulus* CK-15 against *S. sclerotiorum*. Indeed, Yang et al. demonstrated the biocontrol potential of wuyiencin in vitro through its: (1) direct antifungal effect by inhibiting hyphal growth, modifying mycelium morphology, and causing cell plasma leakage; (2) inhibition of the pathogen’s diffusion by affecting the expression of virulence factors during the infection; (3) reduction of the initial source of the inoculum by inhibiting the production and germination of sclerotia. In addition, a first step towards in planta control using wuyiencin has been made on detached soybean leaves [49].

Numerous fungal species of biocontrol interest against *S. sclerotiorum* have also been studied. Among these, different *Trichoderma* spp. produce volatile and non-volatile antimicrobial metabolites with various activities against *S. sclerotiorum* [38], including the non-volatile metabolite harzianic acid. In vitro tests have shown that 2-hydroxy-2-[4-(1-hydroxyocta-2,4-dienylidene)-1-methyl-3,5-dioxopyrrolidin-2-ylmethyl]-3-methylbutyric acid, also known as iso-HA, a diastereoisomer of harzianic acid, can inhibit the growth of *S. sclerotiorum* [38].

Tomah et al. demonstrated the antifungal activity of silver nanoparticles (AgNPs) synthesized by *T. virens* HZA14, which also produces the antibiotic gliotoxin; both are antagonistic against *S. sclerotiorum*. The biosynthesized AgNPs showed a strong hyphal growth inhibition, a reduction in sclerotia formation, and sclerotial mycelium germination under in vitro conditions. Characterizations of AgNPs synthesized by *T. virens* HZA14 revealed an interaction pattern between AgNPs and other metabolites, in particular with gliotoxin, leading to strong pathogen inhibition [46].

*Coniothyrium minitans*, another fungus with biocontrol interest, has been shown to be antagonistic against *S. sclerotiorum* in several host plants such as sunflower, lettuce, cucumber, beans, and rapeseed [55,56]. Two commercially available biocontrol products developed with *C. minitans* propagules are currently available, Contans^®^ and KONI^®^ [55]. Among their modes of action, the production of the antibiotic macrosphelide A was associated with fungal growth inhibition [55]. Confrontational tests with *S. sclerotiorum* showed that the pathogen was consistently inhibited at a high level by *C. minitans*, suggesting the implication of inhibitory metabolites [55].

To date, research on the biocontrol of *S. sclerotiorum* using antibiosis has been mostly tested in vitro. Experiments that have clearly demonstrated in planta pathogen growth inhibition by antibiosis (mostly under growth chamber conditions) have mainly used *Pseudomonas* spp. as biocontrol agents, more specifically members of the *P. fluorescens* group [41,42,43]. HCN production by *Pseudomonas* strains has been shown to be a significant contributor to the in planta biocontrol of *S. sclerotiorum* [41,42]. Other molecules (Table 1) have also proven to be effective against *S. sclerotiourm*, but have so far only been tested under in vitro conditions. The antagonistic activity of these molecules should be tested in planta to determine their ability to protect plants against white mold development.

Most in planta-conclusive results using antibiosis have been obtained using aerial inhibition by spraying antagonistic *Pseudomonas* spp. and *S. sclerotiorum* ascospores on leaves or petals [41,43,44]. Indeed, the aerial infection caused by *S. sclerotiorum* ascospores is central to white mold disease development. However, as discussed earlier, underground plant infection by *S. sclerotiorum*’s sclerotia also represents another important avenue used by the pathogen to cause disease development. To our knowledge, biocontrol specifically targeting *S. sclerotiorum* underground plant infection has not been studied so far, but represents a viable control strategy that should be explored.

### 10.2. Induced Systemic Resistance (ISR)

ISR is a mechanism that enhances plant defenses to mobilize cellular defense responses during pathogen attack, and are typically activated by microbe-associated molecular patterns (MAMPs) and by the release of volatile organic compounds (VOCs) [57,58]. MAMPs produced by PGPRs have a role in triggering phytohormone signaling pathways that enhance disease resistance and are recognized by the plants’ molecular pattern recognition receptors [59,60]. These receptors typically recognize the structure of MAMPs and trigger ISR [60]. There are several MAMPs, including cell wall components such as flagellin and lipopolysaccharide (LPS), as well as LPs [61]. MAMPs and VOCs are produced by plant-growth-promoting rhizobacteria (PGPRs) such as *Pseudomonas* sp. and *Bacillus* sp., as well as fungi such as *Thrichoderma* sp., and they trigger jasmonic acid (JA) and ethylene (ET) signaling pathways involved with ISR. JA and ET signaling pathways are critical in the regulation of ISR [62,63]. Some studies have also reported that the salicylic acid (SA) signaling pathway, activated by the release of defense elicitors by a pathogen, may also trigger ISR [62,64,65]. In both cases, there is an activation of latent defense mechanisms that are expressed locally at the site of induction and a signal is then translocated systemically in the plant following an infection by a pathogen [62]. Plants that perform ISR will show increased expression of genes primarily regulated by these hormones, a phenomenon called “defense priming” [66]. The production of these hormones leads to fighting pathogen attacks more rapidly and/or more strongly by activating cellular defenses upon invasion, making structural and biochemical changes that ultimately lead to an increased level of resistance [62,63]. There are several defense-related genes that are involved in ISR to protect plants from *S. sclerotiorum* infection, including *PR1*, *PR2*, *PR3*, *SOD*, *PPO*, *PAL*, *GST*, *HMGR AOC3*, *PDF1.2*, *ERF2,* and *MPK3* [67,68,69]. Overexpression of these genes triggers the production of defense-related proteins [68]. The increase in chitinases, B-1,3-glucanases, peroxidases, lipoxygenases, superoxide dismutases, phenylalanine lyases, and polyphenol oxidase phytoalexins, as well as the formation of protective biopolymers such as lignin, callose, and hydroxyproline, are among the defense mechanisms expressed following ISR [68,70]. ISR has been highlighted in several studies as the primary mechanism responsible for biocontrol activity against *S. sclerotiorum*, as summarized in Table 2.

Zhang et al. studied the resistance mechanisms in a tripartite interaction between *Trichoderma hazarium* T-aloe, *S. sclerotiorum,* and soybean plants. RT-qPCR analysis of the expression of the genes *PR1* (unknown enzyme activity, antifungal), *PR2* (β-1,3-glucanase), and *PR3* (chitinase) was performed to determine the induction of *T. harzianum*-mediated transduction pathways dependent on SA, JA, or ET [69]. The expression of all three genes in soybeans was highest in the *T. harzianum*-inoculation treatment when followed by *S. sclerotiorum.* The control treatment showed no target gene expression [69]. Their results suggest that ISR induced by *T. harzianum* in soybean plants involves the SA and JA/ET pathways [69]. Alkooranee et al. demonstrated that ISR triggered by *T. harzianum* TH12 in *Brassica napus* and *Raphanus alboglabra* overexpressed three different resistance genes, *AOC3*, *PDF1.2,* and *ERF2*, all markers of the JA/ET pathways. The induction of the JA/ET-dependent defenses decreased the symptoms in infected leaves also treated with TH12 [67].

Aggeli et al. evaluated the plant defense-triggering activity of *Arthrobacter* sp. FP15 by quantifying the relative expression of the SA, JA, and ET-dependent plant defense marker genes *PR1*, *LOX,* and *ERF1*. The plants treated with FP15 featured reduced and delayed symptoms’ development when compared to the control; the first symptoms of plants treated with FP15 appeared 11 days post-inoculation when compared to the control treatment, where 38% of plants showed symptoms 7 days post-inoculation [74]. They also demonstrated an upregulation of *LOX* and *ERF1* gene expression in the later stages of infection, compared to the control treated with *S. sclerotiorum* only [74]. JA/ET-dependent defenses were induced throughout the experiment [74].

As previously mentioned, some *B. amyloliquefaciens* are biocontrol agents that use antibiosis against *S. sclerotiorum.* In addition to exhibiting direct antifungal activity, fengycin produced by *B. amyloliquefaciens* FZB42 appears to regulate the expression of plant defense-related genes involved in ISR [68]. Fungicin-treated tomato plants significantly regulated the expression of six defense-related genes, including *SOD*, *PPO*, *PAL*, *GST*, *HMGR,* and *MPK3*, contributing to the protection of the plant from infection by *S. sclerotiorum*. Mitogen-activated protein kinases (MAPKs) have been shown to play an important role in signal transduction in response to hormones and environmental stresses, and some MAPK family members have been implicated in plant defense as components of defense signaling pathways [75]. As mentioned above, flagellin MAMP can induce ISR. In *Arabidopsis thaliana*, the bacterial flagellum-derived flg22 peptide can trigger the activation of AtMAPKs such as AtMPK3, which seems to play beneficial roles in plant immunity [76]. Wang et al. demonstrated that BnaMPK3, an MPK3 ortholog in *B. napus* induced by JA and the biosynthesis precursor of ET, plays an important role in the activation of ET defense signaling against *S. sclerotiorum* by using both gain- and loss-of-function approaches. The resistance to *S. sclerotiorum* is significantly enhanced by the over-expression of *BnaMPK3* in *B. napus* and *Nicotiana benthamiana* [76]. In addition, a candidate gene association analysis was used to validate the contribution of genomic loci for the resistance against *S. sclerotiorum* [76]. The results suggested that the resistance to *S. sclerotiorum* is a trait with a very complex genetic basis that is determined by multiple minor quantitative trait loci (QTLs) [76].

Research on ISR against *S. sclerotiorum* is still in its infancy. The functioning of this biocontrol mechanism is not yet completely characterized in different plant species. The two main biocontrol agent genera studied so far for their capacity to induce a plant systemic resistance response against white mold, *Trichoderma* spp. and *Bacillus* spp., have been mostly tested on one single plant species, *Brassica napus* [67,68,69,71,75]. Additionally, the real contribution of ISR to controlling *S. sclerotiorum* in the field remains to be better characterized. Most studies on ISR have so far focused on studying the phytohormonal signaling pathways and the translocation of latent defense mechanisms systemically in the plant following pathogen infection. Unfortunately, the results obtained so far on hormone signaling pathways have, in some cases, been contradictory [62,67,69,72,74]. Many unknown variables that make the characterization of the mechanism itself difficult have been reported in these studies. In the context of white mold, and to have a better chance of controlling *S. sclerotiorum* in the field, future studies on ISR should consider inoculating the pathogen not only on leaves but also in the soil to better understand how ISR can contribute to the biocontrol under these different, yet complementary, plant colonization patterns used by the pathogen in real life conditions.

### 10.3. Mycoparasitism

Another effective biocontrol mechanism against *S. sclerotiorum* is mycoparasitism and is defined as a biotrophic interaction between an organism and a fungus in which the organism benefits at the expense of the fungus [77]. This mechanism is the most important form of antagonism that directly affects the pathogen’s mycelium through physical contact [78]. Mycoparasitism can be divided into four main stages: (i) the chemotropic growth of the mycelium from the fungal antagonist to the phytopathogenic fungus, (ii) recognition, (iii) direct attachment and degradation of the cell membrane, (iv) penetration of host fungal cells [79]. In the case of *S. sclerotiorum*, mycoparasitism can directly affect the mycelium, sclerotia, and apothecia through physical contact [80]. Some fungal species, mostly belonging to the genera *Coniothyrium* spp. and *Trichoderma* spp., have demonstrated biocontrol activity through mycoparasitism, as shown in studies listed in Table 3.

*C. minitans* can parasitize *S. sclerotiorum*’s sclerotia and produce compounds that inhibit its growth [101]. It has been proven to successfully control diseases caused by *S. sclerotiorum* in the field [102]. In order for *C. minitans* to be able to kill its host and gain access to nutriments, it must penetrate the sclerotia’s cell membrane [103]. Hydrolytic enzymes produced by *C. minitans*, such as chitinases, have been shown to play an active role in this crucial stage of mycoparasitism, in addition to playing a role in remodeling its cell membrane as it grows inside the phytopathogen [37,103]. Zhao et al. assembled and analyzed the genome and transcriptome of the *C. minitans* strain ZS-1 during its early interaction with *S. sclerotiorum* to better understand parasitism [104]. Expressed genes involved in host defense responses were detected, including CWDEs, transporters, secretory proteins, and secondary metabolite productions. Seventeen DEGs of fungal CWDEs were up-regulated during parasitism [104]. Shared and unique characteristics of the major facilitator superfamily (MFS), ABC transporter proteins, secretory proteins, and secondary metabolite biosynthesis gene clusters were identified as being involved [104].

A model describing the interaction between *C. minitans* and *S. sclerotiorum* suggests that *C. minitans* triggers the production of antifungal compounds to inhibit the growth of *S. sclerotiorum* when the pH decreases by the production of oxalic acid by *S. sclerotiorum* [105]. *C minitans* begins its parasitic life stage by secreting CWDEs when the pH returns to normal [105]. The *S. sclerotiorum* response was, however, neglected in this model. Zhao et al. demonstrated that many genes associated with MFS transporters, ABC transporters, effector-like proteins, and secondary metabolites were significantly up-regulated during the early stages of interaction, suggesting that these genes play a role in the parasitism of *S. sclerotiorum*.

Rajani et al. demonstrated the mycoparasitism of *S. sclerotiorum* by three species of *Trichoderma* sp. using a scanning electron microscope. The three species (*T. longibrachiatum* MK425639 and MK751759, *T. harzianum* MK751758, and *T. pleuroti* MK751757) grew densely along the mycelium of *S. sclerotiorum*, often branching abundantly and wrapping around it [84]. The invasion of *S. sclerotiorum*’s mycelium by the endophytes was suggested by observing the extensive deformation of each fungus [84].

The overall number of studies that directly investigated the role of mycoparasitism in the control of *S. sclerotiotum* in vitro remains small. There are even less studies that were performed in planta, whether in growth chambers or under field conditions. Among these, *Trichoderma* spp. has been the most widely used genus in the context of mycoparasitism. Studies that have demonstrated the penetration of sclerotia by the biocontrol agent appears as the most promising [85,86,87,89,90,93,95,96,97,98,99]. More specifically, the degradation of melanin in sclerotia, which acts as a strong protection against lysis in nature, should be further studied. A better understanding of the mechanisms allowing sclerotia penetration and the characterization of the conditions required for it to happen will require additional experimentation.

### 10.4. Hypovirulence

A very promising approach for achieving the effective biocontrol of infections caused by various fungal pathogens, including *S. sclerotiorum*, is the use of hypovirulent strains of the pathogenic fungus itself. Hypovirulence refers to a decreased ability of certain isolates of pathogenic fungi to infect, colonize, decrease viability or vitality, or reproduce on susceptible host plants [106,107]. Hypovirulence is commonly associated with fungal infection by mycoviruses, typically of the genera *Mitovirus* or *Hypovirus* [106]. These non-encapsulated viruses contain small, positive sense, single-stranded RNA genomes and the infection of target fungi with these viruses is associated with the accumulation of dsRNA elements and a decrease in the pathogenicity of the infected host [106]. While most hypovirulence-associated viral taxa are RNA viruses, one of the most promising viruses is a dsDNA virus, SsHADV-1 (*S. sclerotiorum* hypovirulence-associated DNA virus 1) [108,109]. This virus was isolated from a hypovirulent strain, DT-8, of *S. sclerotiorum*. The application of strain DT-8 to *B. napus* prior to the exposure to a virulent strain of the pathogen greatly decreased the resultant severity of Sclerotinia stem rot on the treated plants, with a concomitant yield increase when compared to untreated plants [109]. Moreover, Qu et al. demonstrated that treatment with DT-8 induced effects on the epiphytic microbial taxa (bacterial and fungal) in and around the stem rot lesions when compared to untreated plants, with a decreased abundance of potential plant pathogens in treated plants. This suggests that “bio-priming” with a hypovirulent strain of *S. sclerotiorum* may partially mediate its protective phenotype through effects on the microbiota of the host plants, by encouraging the appearance of a diverse network of interconnected microbes that can help the host plant resist infection [109]. The interaction of *S. sclerotiorum* with plant hosts can take different forms, including endophytic, non-pathogenic growth in certain cereal crops that can have a protective effect against infection with other fungal plant pathogens such as *Fusarium* sp. [110]. Alternatively, infection with a virus-infected hypovirulent strain such as SsHADV-1 can offer protection against infection in this same context. These alternative, host-dependent modes of interaction with the host plant, either pathogenic or mutualistic (endosymbiotic), have been termed “schizotropism” to reflect the host-dependent mode of interaction displayed by certain fungi, including *S. sclerotiorum* [110]. Schizotropism manifests as a broad-spectrum pathogen of some plants being a suitable biocontrol agent in another group of plants, offering a possible mode of application of pathogenic fungi as biocontrol agents in certain situations. In addition, hypovirulent isolates of pathogenic fungi can offer protection against infection by virulent strains of other fungal species; for example, the hypovirulent isolate QT5-19 of *Botrytis cinerea* displayed increased competitive saprophytic activity in vitro when compared to virulent strains of both *B. cinerea* and *S. sclerotiorum*, and was effective in suppressing an infection of susceptible plants by both fungi [111]. These considerations emphasize the complexity of host–pathogen interactions and the necessity of fully understanding the nature of these relationships in order to maximally exploit their potential, to mitigate the devastating effects of *S. sclerotiorum* on crop species.

## 11. An Amalgam of Biocontrol Agents

The mechanisms involved in biocontrol that determine the success or failure of biocontrol agents are complex and are largely affected not only by the plant but also by environmental conditions [112,113]. So far, the success of biocontrol observed in the field has been limited because of environmental variability and a lack of biocontrol mechanisms understanding [113]. As shown above, the majority of studies performed to date have investigated a specific biocontrol mechanism [114]. Yet, a biocontrol agent may use several biocontrol mechanisms [114] or multiple biocontrol agents relying on different control mechanisms can be deployed simultaneously. Since environmental conditions can influence the different biocontrol mechanisms involved differently, one mechanism may compensate for another less effective one under a given condition [114]. The use of multiple biocontrol agents with multiple control mechanisms could also counteract changing environmental conditions [112]. However, proper population dynamics studies should be performed, as the combination of two biocontrol agents placed in the same niche may lead to an antagonistic interaction against each other [115]. Delivering biocontrol agents in different niches might also be an interesting approach to use [115]. In this case, the biocontrol agents never compete with each other. This strategy has been demonstrated in the biocontrol of other ascomycetes resembling *S. sclerotiorum* [115]. Further research will however still be needed to determine under which heterogeneous conditions the combination of biocontrol agents can result in a synergistic interaction that reduces the viability of the pathogen [115]. To our knowledge, no studies have so far reported on the biocontrol of *S. sclerotiorum* using synergistic biocontrol agents. Future studies could therefore use biocontrol agents that have been shown to inhibit the growth of *S. sclerotiorum* and combine them to see if their combined inhibition effect is greater.

## 12. Conclusions

The accumulation of *S. sclerotiorum*’s sclerotia in agricultural soils due to the continued use of susceptible cultivars increases losses worldwide. The wide host range and the long-term survival of sclerotia leads to difficult, inconsistent, and uneconomical management. The economical and durable management of white mold should thus be based on an integration of a variety of control methods related to avoidance, exclusion, eradication, protection, and therapy. Considering the cost and limited efficacy of synthetic fungicides, biological control should play an increasing role in integrated management of white mold.

In this review paper, we highlighted desirable biocontrol traits that have demonstrated efficacy against *S. sclerotiorum* and white mold. No single trait identified so far seems sufficient to properly control the disease and, in light of the data available, we believe that a combinatory biocontrol approach should be considered. Although antibiosis and induced systemic resistance have been the most-studied biocontrol mechanisms against white mold, mycoparasitism and hypovirulence also show promise. Developing inoculants containing compatible biocontrol agents, yet relying on different biocontrol mechanisms, appears to be as a promising strategy. Different *S. sclerotinium*’s life cycle stages can be targeted, including soil applications to prevent sclerotia germination, mycelium growth, and infection. Reducing pathogen loads directly in soil appears especially promising in the context of *S. scleroriorum*, which in turn would also indirectly reduce ascospores formation and release.

However, as for all control methods, a successful strategy should be based on a deep knowledge of the epidemiology and ecology of *S. sclerotiorum*, in line with the modes of action of biological control agents. Biocontrol techniques that use microbial agents to at least minimize the infection of *S. sclerotiorum* are promising but, as shown in this paper, information is still missing, such as a better understanding of the biocontrol mechanisms involved and the conditions required to express these mechanisms to their full potential. Further research on both the biology of *S. sclerotiorum* and the biocontrol mechanisms involved, including the development of combinatory biocontrol approaches, is essential for the development of integrated control measures. A better knowledge of the mechanisms involved in biocontrol will not only deepen our understanding of plant–microbe and microbe–microbe interactions, but will also exploit the different modes of action of beneficial microbes in the field.

## Figures and Tables

**Table 1:** Antimicrobial molecules inhibiting *S. sclerotiorum* through antibiosis.

Molecules	Species	Strains	Experiment Conditions	Plants	References
Bacillomycin D	*Bacillus amyloliquefaciens*	SQR9	B		[40]
Bacillibactin	*Bacillus amyloliquefaciens*	SQR9	B		[40]
Hydrogen cyanide	*Pseudomonas brassicacearum*	DF41	A and B	Canola	[41]
	*Pseudomonas cholororaphis*	PA-23	A and B	Lettuce	[42]
Pyrrolnitrin	*Pseudomonas chlororaphis*	PA-23	A	Canola	[43]
Iturin A	*Bacillus velezensis*	KRF-001	A	Lettuce	[44]
Surfactin	*Bacillus amyloliquefaciens*	SQR9	B		[40]
Phenazines-1-carbolylic acid	*Pseudomonas cholororaphis*	PA-23	A	Canola	[43]
Sclerosin	*Pseudomonas brassicacearum*	DF41	B		[45]
Silver-nanoparticles (Gliotoxin)	*Trichoderma virens*	HZA14	B		[46]
2-undecanone	*Bacillus velezensis*	VM11	B		[47]
Benzothiazole	*Bacillus velezensis*	VM11	B		[47]
1,3-butadiene	*Bacillus* sp.		B		[47]
N,N-dimethyldodecylamin	*Bacillus* sp.		B		[47]
Pentadecane	*Bacillus* sp.		B		[47]
IR-(+)-α-pinene	*Bacillus* sp.		B		[47]
Albocycline	*Propionicimonas* sp.	ENT-18	B		[48]
Wuyiencin	*Streptomyces albulus*	CK-15	B		[49]

A: Greenhouse/Growth Chamber; B: In vitro conditions.

**Table 2:** Microbial strains inhibiting *S. sclerotiorum* through induced systemic resistance.

Species	Strains	Plants (Hosts)	References
*Trichoderma harzianum*	T-aloe	Soybean	[69]
	TH12	*Brassica napus*	[67]
	TH12	*Brassica napus*, *Raphanus oleracea*	[71]
*Trichoderma viride*	TV10	*Brassica napus*, *Raphanus oleracea*	[71]
*Paenibacillus alvei*	K165	Lettuce	[72]
*Bacillus amyloliquefaciens*	FZB42	Tomato	[68]
*Bacillus thuringiensis*	4M1, 4I4, 4F5, 4CC1, 4BM1, 4B1, 4BU1, 4D19, 4F2,4J3, 4O1, 4AL1, 4AP1, 4BD1, 4D3, 4XX1, 4AZ1	*Brassica c* *ampestris*	[73]
*Arthrobacter*	FP15	Lettuce	[74]

**Table 3:** Microbial strains inhibiting *S. sclerotiorum* with mycoparasitism through microscopy.

Species	Strains	Experiment Conditions	Plant (Host)	References
*Trichoderma harzianum*	BAFC	B and C	Sunflower, Lettuce	[81]
	KucF010	B and C	Tomato, Eggplant, Squash	[82]
	8	C		[83]
		C		[84]
*Trichoderma* spp.	T12-9	C		[85]
*Trichoderma atroviride*	PTCC5220	C		[83]
*Trichoderma longibrachiatum*	PTCC5140	C		[83]
	2	C		[84]
*Trichoderma pleuroti*		C		[84]
*Trichoderma virens*	I10	C		[86]
*Trichothecium roseum*	TR-4, TR-6	C		[87]
*Gliocladium virens*		C		[88]
	G20	C		[89]
		C		[90]
*Gliocladium* spp.	G21-3	C		[85]
*Gliocladium roseum*	67-1	C		[91]
*Coniothyrium minitans*		C		[92]
		C		[89]
	IMI 134523	C		[93]
	A2960	C		[94]
		C		[90]
	CON/M 91-08	C		[95]
*Microsphaeropsis ochracea*	P130A	C		[95]
*Aspergillus terreus*		C		[96]
*Fusarium* spp.	D6-15	C		[85]
*Fusarium oxysporum*	S6	C		[97]
*Sporidesmium sclerotivorum*	CS-5	C		[98]
		C		[99]
		A		[100]
*Teratosperma oligocladum*	TO-2	C		[98]
*Dictyosporium elegans*		C		[90]

A: Field conditions; B: Greenhouse/Growth Chamber; C: In vitro conditions.

## Data Availability

Not applicable.
